# Endothelial genetic deletion of CD147 induces changes in the dual function of the blood‐brain barrier and is implicated in Alzheimer’s disease

**DOI:** 10.1111/cns.13659

**Published:** 2021-05-13

**Authors:** Hao Wang, Jian‐Jun Lv, Yu Zhao, Hao‐Lin Wei, Tian‐Jiao Zhang, Hai‐Jiao Yang, Zhi‐Nan Chen, Jian‐Li Jiang

**Affiliations:** ^1^ Department of Cell Biology National Translational Science Center for Molecular Medicine Fourth Military Medical University Xi’an China

**Keywords:** Alzheimer's disease, blood‐brain barrier, CD147, neurodegenerative disease, transcriptome

## Abstract

**Aims:**

The blood‐brain barrier (BBB) is a specialized and indispensable structure in brain blood vessels that is damaged during Alzheimer's disease (AD). CD147 is expressed on the BBB and deeply engaged in the AD pathological process. In this study, we aimed to provide a better understanding of the roles of CD147 in BBB function in health and neurodegenerative disease.

**Methods and Results:**

We measured CD147 expression in mouse brains and demonstrated that CD147 is exclusively expressed in brain endothelial cells (BECs), and its expression decreases with age. After constructing endothelial‐specific CD147 knockout mice, we performed RNA‐sequencing on BECs isolated from mice of different ages as well as a range of database analyses. We found that endothelial CD147 is essential for the dual functions of the BBB, including barrier maintenance and transporter regulation. This study also shows that CD147 plays a pivotal role in neurodegenerative diseases, particularly in AD.

**Conclusions:**

Our findings suggested that targeting CD147 in BECs may represent a novel therapeutic strategy, which promoted the design of future experimental investigations and the mechanistic understanding of neurodegenerative diseases.

## Graphical abstract

Mouse primary brain endothelial cells isolated from endothelial‐specific CD147 knockout mice of different ages were performed RNA‐sequencing. CD147 is essential for the dual functions of the blood‐brain barrier, including barrier maintenance and transporter regulation, and neurodegenerative diseases, particularly Alzheimer's disease.

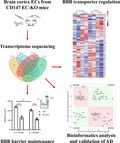

## 1 INTRODUCTION

Alzheimer's disease (AD) is a progressive and age‐dependent neurodegenerative disease that contributes to 60%–70% of dementia worldwide. Although AD etiology is consistently being explored, a definitive conclusion has still not been reached. The classic theories involve Aβ plaques and Tau neurofibrillary tangles, which are commonly accompanied by APP or PSEN1/2 mutations. In addition, ApoE4 is one of the biggest risk factors for sporadic AD and is also involved in Aβ pathology.^1^, ^2^ Furthermore, neuroinflammation and microglial overactivation also play important roles in promoting AD.^3^, ^4^

Several lines of evidence have suggested that the blood‐brain barrier (BBB) is damaged in the course of AD.^5^ The BBB is a specialized structure that maintains CNS homeostasis and comprises BECs, PCs, the endfeet of astrocytes, and the vascular basement membrane.^6^ When the BBB interacts with surrounding neurons, microglia, and other brain components, they are collectively referred to as a neurovascular unit.^7^, ^8^ The BBB is orchestrated by a dynamic balance between its two functions: preventing pathogens and toxins from entering the parenchyma and transporting nutrients and metabolites. Notably, the paracellular barrier is primarily determined by BECs, which are effectively sealed with tight junctions (TJs). While several methods have been developed to deliver drugs across the BBB through transporters on BECs, such as receptor‐mediated transcytosis and carrier‐mediated transport,^9^, ^10^, ^11^, ^12^, ^13^ enormous difficulties still need to be overcome. A more elaborate elucidation of the BBB can help us better understand the biological characteristics and develop effective drugs for CNS disease.

CD147 is a member of the immunoglobulin superfamily. This transmembrane glycoprotein is encoded by the *BSG* gene and is also known as extracellular matrix metalloproteinase inducer for its features. CD147 exerts pivotal effects on malaria invasion,^14^ *Neisseria meningitidis* adhesion,^15^ and inflammatory diseases.^16^ CD147 was shown to be a BEC‐specific molecule in chicks in 1986,^17^ and recent research has indicated that CD147 is highly expressed on mouse BECs and could be a potential target mediating antibody transport across the BBB.^18^ In addition, CD147 expression is increased in a mouse model of ischemic stroke, resulting in impaired brain function through MMP production.^19^ CD147 colocalizes with monocarboxylate transporters (MCT1, expressed on the BBB), and serves as an essential chaperone colocalized on the plasma membrane.^20^ Moreover, CD147 inhibition can suppress total and exosomal Aβ_42_ production,^21^ which may contribute to the formation of amyloid plaques in AD. However, the roles that CD147 might play in BBB integrity and AD have not been fully explored.

In this study, we demonstrated that CD147 is specifically expressed on mouse BECs under normal conditions and that there is a marked decrease in its expression with age. Moreover, we constructed EC‐specific *CD147* knockout mice and performed RNA‐sequencing with different genotypes and ages. We suggest that CD147 is essential for the maintenance of BBB integrity and the regulation of carriers and receptors and provide profound insights into strategies for studies of neurodegenerative diseases, particularly AD.

## 2 MATERIALS AND METHODS

### 2.1 Animals

*APP*/*PS1* double transgenic mice over 18 months old and their WT littermates were used for experiments. To generate endothelial cell‐specific *CD147*‐knockout (EC‐KO) mice, *H11*‐*Tek^cre^* ^/^ *^cre^* mice were crossed with *CD147^f^* ^/^ *^f^* mice constructed by our lab.^22^ *H11*‐*Tek^cre^* ^/+^ *CD147^f^* ^/^ *^f^* (named EC‐KO) and *H11*‐*Tek* ^+/+^ *CD147^f^* ^/^ *^f^* (named WT) mice of two age groups (8–12 weeks old and 6–7 months old) were used after mating for several generations. All mice were maintained in specific pathogen‐free conditions at a controlled temperature (22–25°C) with an alternating 12‐h light/dark cycle. All animal experiments in this study were carried out in accordance with the Animal Research: Reporting In Vivo Experiments (ARRIVE) guidelines^23^ and were approved by the Animal Care and Use Committee of the Fourth Military Medical University.

### 2.2 Immunofluorescence

For tissue staining, mouse brains were fixed in 4% paraformaldehyde after perfusion, dehydrated in sucrose, and embedded in optimal cutting temperature compound. Tissue slices were blocked with 10% BSA and incubated with primary antibodies overnight at 4°C. The following antibodies were used, including rabbit NeuN polyclonal antibody (1:500, cat. ABN78, Millipore), mouse GFAP monoclonal antibody (1:200, cat. 3670, Cell Signaling Technology), rabbit MBP polyclonal antibody (1:200, cat. ab40390, Abcam), rabbit Iba1 monoclonal antibody (1:1000, cat. ab178846, Abcam), goat CD31 polyclonal antibody (1:200, cat. AF3628, R&D), and PE anti‐human CD147 antibody (1:100, cat. 123705, BioLegend). The slices were incubated with Alexa Fluor 488‐labeled donkey anti‐rabbit secondary antibody (1:200, cat. A21206, Thermo Scientific), Alexa Fluor 488‐labeled donkey anti‐mouse secondary antibody (1:200, cat. A21202, Thermo Scientific), or FITC‐AffiniPure rabbit anti‐goat IgG (H+L) antibody, (1:100, cat. 305–095–003, Jackson ImmunoResearch Labs). The nucleus was stained with DAPI and images were captured by a fluorescence microscope (Olympus).

For cell immunofluorescence, cells were fixed with 4% paraformaldehyde, permeabilized with 0.02% Triton X‐100, blocked with 10% BSA, and incubated with primary including CD31 polyclonal antibody and rabbit CD147 monoclonal antibody (1:200, cat. ab188190, Abcam). The dishes were incubated with Alexa Fluor 555‐labeled donkey anti‐goat secondary antibody (1:200, cat. ab188190, Thermo Scientific) and Alexa Fluor 488‐labeled donkey anti‐rabbit secondary antibody and DAPI. Images were obtained with an A1R‐A1 confocal laser microscope system (Nikon).

### 2.3 Immunohistochemistry

Upon perfusion, mouse brains were fixed, dehydrated, and embedded in paraffin. Tissue slices were stained with primary antibodies, including rabbit CD147 monoclonal antibody (1:3000, Abcam), rabbit beta Amyloid (1‐42) polyclonal antibody (1:500, cat. GTX134510, GeneTex), rabbit CD45 polyclonal antibody (1:500, cat. 20103‐1‐AP, Proteintech), and rabbit Iba1 monoclonal antibody (1:2000), followed by HRP‐conjugated secondary antibodies and DAB substrate. Slices were then counterstained with hematoxylin. Images were captured with a microscope (Olympus).

### 2.4 Isolation and culture of mouse brain cortex ECs

Mouse brains were extracted under sterile conditions and the olfactory bulb, cerebellum, white matter, and meninges were removed. The clean cortex was dissected and transferred to cold DMEM containing 2.5 mg/ml type II collagenase. The homogenate was digested for 1 h at 37°C in a hybridization incubator with constant rotation and for an additional hour after 1 mg/ml Collagenase/Dispase was added. The pellet was collected by centrifugation (1000 g, 10 min, 4°C), and the myelin was separated via the addition of 20% BSA in DMEM and centrifugation (1000 g, 20 min, 4°C). The precipitate was resuspended and gently layered on top of 22% (v/v) Percoll and then centrifuged (560 g, 10 min, 4°C, no brake). The microvessel layer was collected and washed in cold DMEM by centrifugation (1000 g, 10 min, 4°C). The cells were seeded in pretreated plates or cell culture inserts coated with type I collagen (0.8 mg/ml, 1 h, RT). The cells were cultured in EGM‐2 medium (Lonza, USA) containing 4 μg/ml puromycin and used for experiments after 5–7 days of culture. The EC samples were pooled from 8 to 10 mice for each group.

### 2.5 In vivo sodium fluorescein (Na‐F) permeability

Mice were injected with 50 mg/ml Na‐F by intravenous injection. After 1.5 h of circulation, the mice were euthanized and perfused with normal saline through the left ventricle. Brains were extracted, and the olfactory bulb and cerebellum were removed. Homogenate was generated using ultrasound in PBS and the absorption of the supernatant was measured at 485 nm using a microplate reader (BioTek) after centrifugation (20,000 g, 30 min, 4°C).

### 2.6 Brain water content

Mouse brains were extracted, and the olfactory bulb and cerebellum were removed. The wet and dry weights of the brains were measured immediately after removal and after drying for 24 h at 100°C, respectively. Brain water content was evaluated to assess microedema and was calculated using the following equation: Water content (%) = (wet weight − dry weight)/wet weight × 100%.

### 2.7 Measurement of TEER and permeability of the BBB model in vitro

To establish an in vitro monocellular BBB model,^24^ primary ECs were cultured in the upper chamber of a Millicell hanging cell culture insert with a 0.4‐μm pore size. After 7 days of culture, a TEER of at least 100 Ω.cm^2^ was measured via an EVOM Volt‐Ohm resistance meter with STX2 chopstick electrodes.

To determine the paracellular permeability of the BBB model, 100 μl of medium with 500 μg/ml of 40‐kDa FITC‐dextran was added to the upper chamber, whereas 1 ml of medium alone was added to the bottom chamber. During culture, 50 μl of medium from the bottom chamber was transferred into a black 96‐well plate with a clear bottom at different timepoints. The fluorescence was measured using a Cytation Imaging Reader (BioTek).

### 2.8 Rhodamine 123 (Rho123) uptake assay

An uptake assay was performed to evaluate ABCB1 transporter function by measuring the intracellular concentration of Rho123. Primary ECs were cultured on 24‐well plates or 35‐mm petri dishes coated with type I collagen (0.8 mg/ml). After 7 days, the cells were incubated with 25 μM Rho123 for 2 h in EGM‐2 medium. The cells were collected and then resuspended in a cell lysis buffer. The fluorescence intensity was measured using a Cytation Imaging Reader and adjusted according to protein concentration (mg). Images were captured via fluorescence microscope.

### 2.9 Expression validation of selected DEGs

Real‐time qPCR and western blot were performed as previously described.^22^ The mRNA levels were normalized against *Gapdh* gene expression. For western blot, primary antibodies were used including goat CD147 polyclonal antibody (1:200, cat. AF772, R&D), rabbit ZO‐1 (TJP‐1) polyclonal antibody (1:1000, cat. 21773–1‐AP, Proteintech), rabbit occluding (OCLN) polyclonal antibody (1:1000, cat. 13409–1‐AP, Proteintech), rabbit SPARCL1 polyclonal antibody (1:50, cat. 13517–1‐AP, Proteintech), rabbit junction adhesion molecule 1 (JAM‐1) monoclonal antibody (1:100, cat. ab52647, abcam), rabbit ABCA1 antibody (1:200, cat. NB400‐105SS, Novus), rabbit INSR polyclonal antibody (1:100, cat. 20433‐1‐AP, Proteintech), rabbit FCGRT polyclonal antibody (1:500, cat. PA5‐79246, Thermo Scientific), and mouse GAPDH monoclonal antibody (1:5000, cat. 60004‐1‐Ig, Proteintech).

### 2.10 RNA‐sequencing and data analysis

RNA‐sequencing was performed by Gene Denovo Biotechnology. Reads were filtered by fastp and then mapped to the reference genome and FPKM values were calculated to quantify gene expression. DEG analysis, correlation analysis of replicates, and GO and KEGG pathway enrichment analyses were also performed. We defined significant DEGs as those with FDR‐adjusted *p* value <0.05, and fold‐change ≥1.5.

### 2.11 Disease‐associated genes and PPI analysis

Neurodegenerative and neurovascular diseases were also evaluated in this study (Table S1). We compared our RNA‐sequencing data with GWAS‐identified genes associated with target diseases, such as AD, PD, and MSA, from the GWAS catalog and selected relevant studies. The intersection was performed in a PPI network via the STRING database.^25^ The minimum required interaction score was set as medium confidence (0.4). The statistical test was performed at ToppGene.

### 2.12 Human brain bulk RNA‐sequencing dataset analysis

The human brain bulk RNA‐sequencing data were obtained from the Aging, Dementia, and Traumatic Brain Injury study dataset of the Allen Institute for Brain Science. Controls were defined based on 111 samples from 30 individuals with the following criteria: diagnosis with “no dementia” and “no history of traumatic brain injury”. Human AD brain samples were classified based on 58 samples from 16 patients with the following criteria: diagnosis with “AD dementia” and “no history of traumatic brain injury”. Comparisons of human brain expression levels (expressed in FPKM) were performed between the two groups by the R package.

### 2.13 Correlation analysis

We downloaded mRNA expression levels (expressed in pTPM) of the human cortex from the GTEx portal. We analyzed 408 samples from three different brain zones, including the anterior cingulate (*n* = 121), frontal cortex (*n* = 129), and cortex (*n* = 158).

### 2.14 Statistical analysis

All experiments were performed independently at least three times. The values are presented as the mean ± SEM. The distribution of the data was assessed using Shapiro–Wilk normality test. For parametric analysis, two‐tailed unpaired Student's *t*‐test or two‐way ANOVA with a post‐hoc Bonferroni's test was used to compare differences between different groups. For non‐parametric analysis, a Mann‐Whitney test was used to compare the values between the two groups. The degree of linearity was analyzed by Spearman correlation. All statistical analyses were performed with GraphPad Prism version 8. Significance was accepted at the level of *p* < 0.05.

## 3 RESULTS

### 3.1 Expression of CD147 on BECs

**FIGURE 1:**
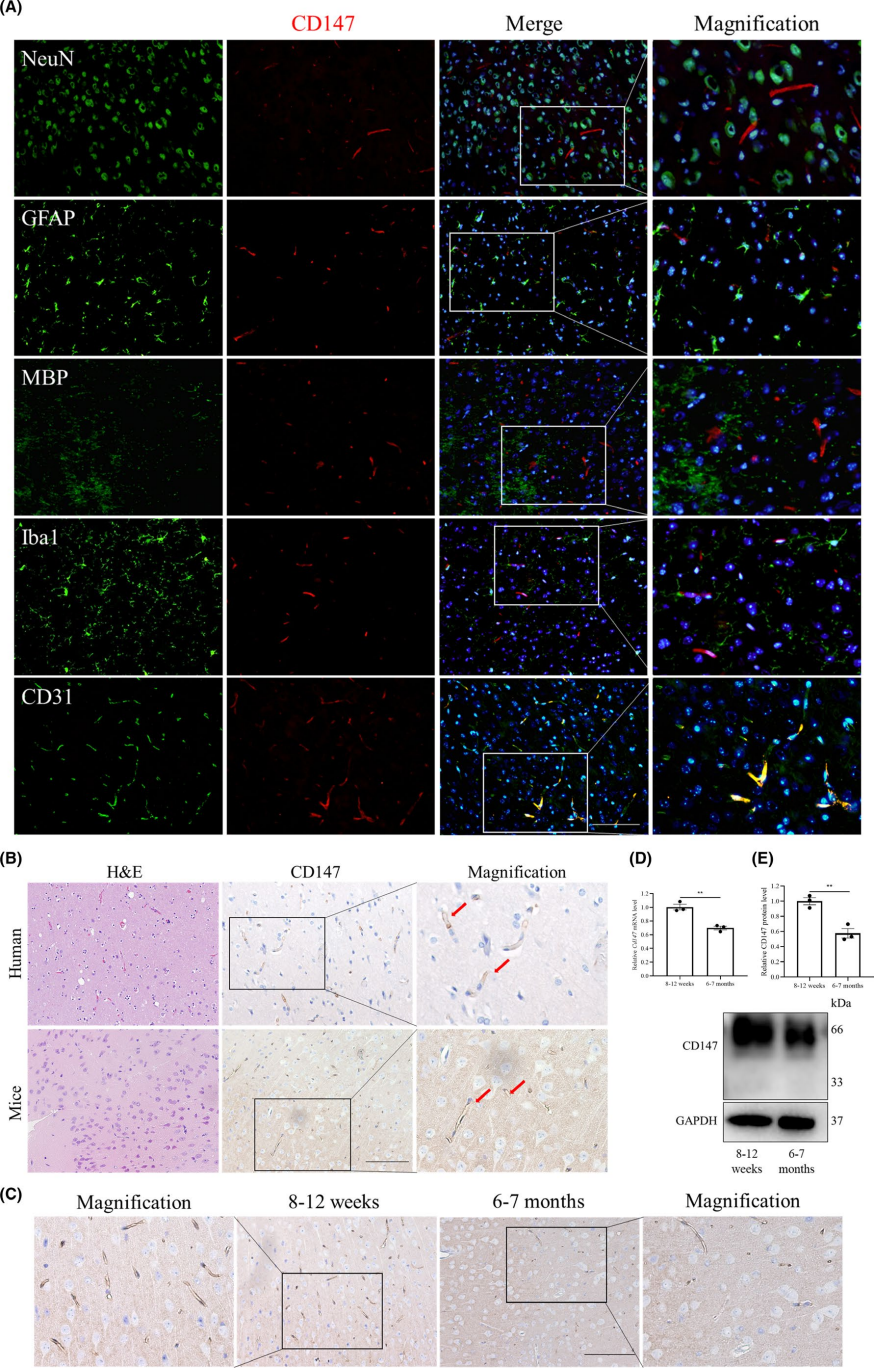
Expression of CD147 on BECs. (A) Brain sections from mice were costained for CD147 (red), NeuN (neuron marker, green), GFAP (astrocyte marker, green), MBP (oligodendrocyte marker, green), Iba1 (microglial cell marker, green), CD31 (endothelial cell marker, green), and nuclei (blue) to evaluate CD147 expression on BECs. Scale bars: 100 μm. (B) H&E and IHC staining of CD147 showed brain features and human and mouse brain vessels stained positively for CD147 (arrowhead). Scale bars: 100 μm. (C) IHC staining of CD147 in 8–12‐week‐old and 6–7‐month‐old mouse brains, Scale bars: 100 μm. (D) RT‐qPCR and (E) WB validated the dynamic CD147 expression in isolated primary BECs from mice between 8 and 12 weeks and 6–7 months of age. Data are presented as the mean ± SEM of *n* = 3 independent experiments. **p < 0.01

To determine the role of CD147 in the brain, we first measured its expression and distribution in 8 to 12‐week‐old mouse brains. We performed double immunofluorescence staining with primary antibodies against CD147, NeuN, GFAP, MBP, Iba1, and CD31. As shown in Figure 1A, CD147 was expressed exclusively on ECs in normal mouse brains. This observation was consistent with the finding that CD147 was expressed on typical strip‐shaped vessels in both human and mouse brain tissues (Figure 1B). To examine CD147 expression changes with age, we probed its expression from mouse brains of two age groups, including 8–12 weeks and 6–7 months old. The immunohistochemical staining results showed that CD147 expression was lower in mice brains aged 6–7 months than in those aged 8–12 weeks (Figure 1C). Similar alterations in CD147 were also observed in mouse primary BECs from the two age groups by RT‐qPCR (Figure 1D) and western blot (Figure 1E). These data suggested that CD147 expression on BECs decreased with age, which probably indicated functional changes in the BBB throughout life.

### 3.2 Construction and identification of CD147 EC‐KO mice

**FIGURE 2:**
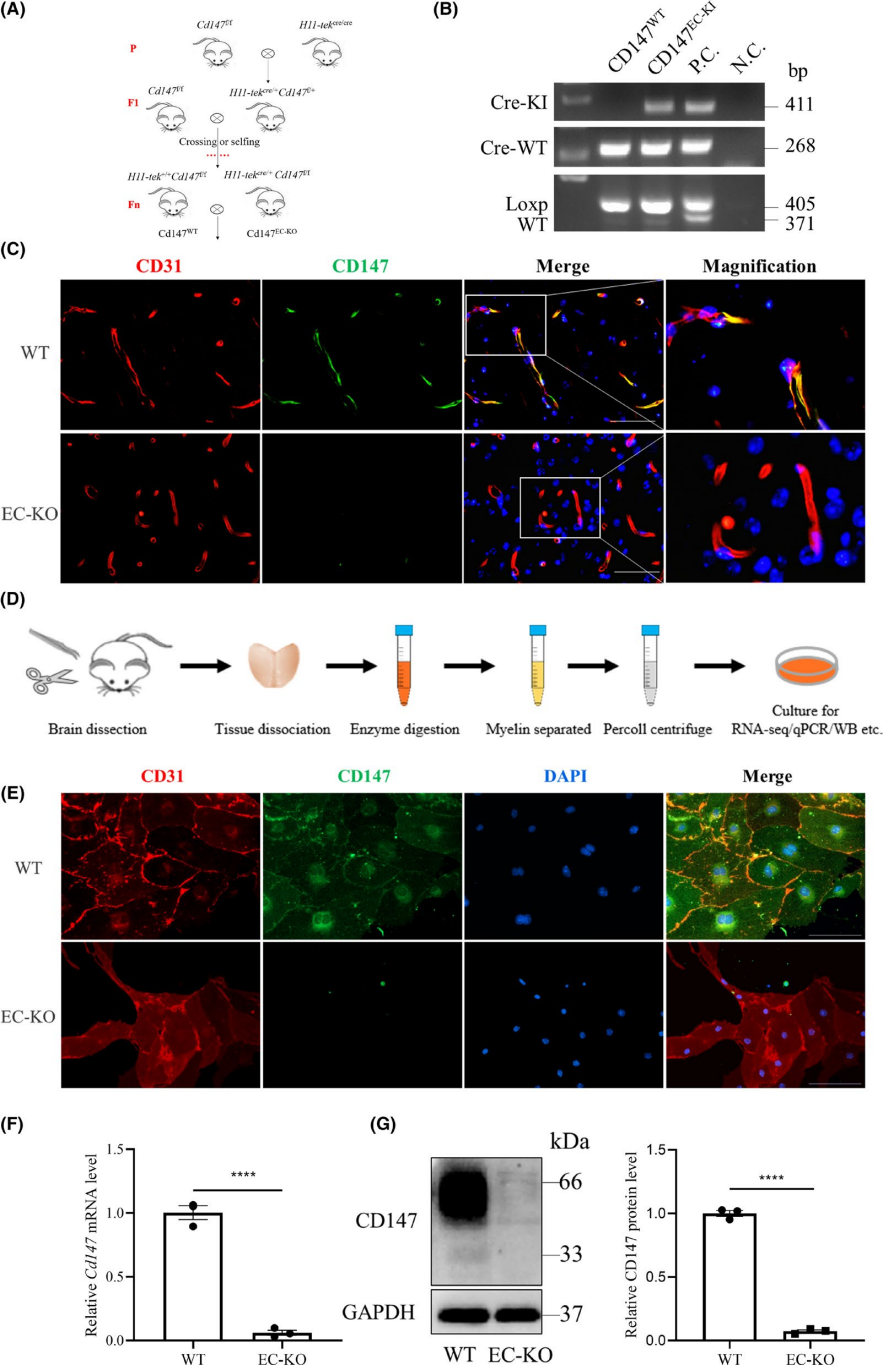
Construction and identification of CD147 EC‐KO mice. (A) Strategies for generating endothelial cell‐specific CD147 knockout mice (EC‐KO) and WT littermates. (B) Representative PCR results of genotype identification. From top to bottom, the bands are Cre‐KI (411 bp), Cre‐WT (268 bp), loxp‐CD147 (405 bp) and wild‐type CD147 (371 bp). (C) Mouse brain slices were costained for CD31 (red), CD147 (green) and nuclei (blue) to verify CD147 knockout efficiency. Scale bars: 50 μm. (D) Diagram of the isolation of primary cortex ECs for further experiments (see the Methods). Validation of isolated cells via (E) immunofluorescence. Scale bars: 100 μm. (F) RT‐qPCR and (G) WB. Data are presented as the mean ± SEM of *n* = 3 independent experiments. ****p < 0.0001

To investigate the effects that CD147 exerts on the BBB, we constructed CD147 EC‐KO mice by using the *Cre*/*LoxP* system. After mating for several generations, we generated EC‐KO and WT mice (Figure 2A). The mouse genotypes were identified by conventional PCR (Figure 2B). Moreover, we ensured that CD147 was specifically knocked out in ECs as indicated by CD147 and CD31 immunofluorescence costaining (Figure 2C). To explore the relationship between CD147 and the BBB, we isolated mouse primary cortex ECs by enzymatic digestion and purification (Figure 2D). The purity of BECs (CD31 positive) was approximately 100%, and CD147 was completely absent in BECs from EC‐KO mice, as shown in Figure 2E. RT‐qPCR (Figure 2F) and western blot (Figure 2G) analyses confirmed the characterization of BECs isolated from EC‐KO and WT mice.

### 3.3 RNA‐sequencing and analysis of mouse primary cortex ECs

**FIGURE 3:**
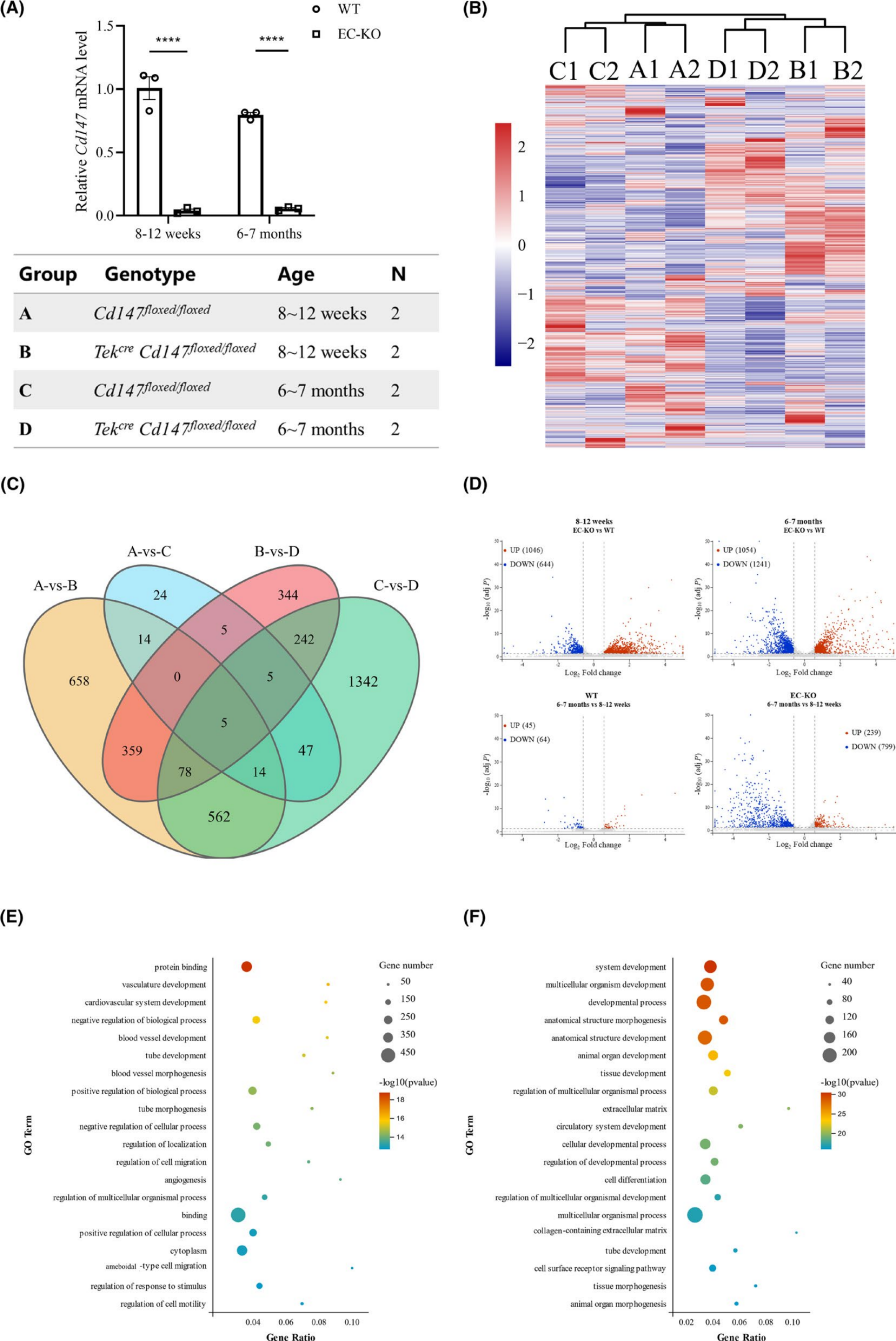
RNA‐seq and analysis of mouse primary cortex ECs. (A) Expression of CD147 was detected before performing RNA‐seq and the groups were based on genotype and age. (B) Hierarchical cluster analysis of all groups showed the reliability of our dataset. (C) Venn diagram of the overlap number of DEGs between comparisons. (D) Volcano plots showing the upregulated (red) and downregulated (blue) DEGs for each comparison. (E, F) Dot plots of GO terms enriched based on the DEGs for genotype (E) and age (F). The CD147 RNA expression level is presented relative to that of GAPDH, and the data are presented as the mean ± SEM of three independent experiments. ****p < 0.0001. DEGs were defined as genes with a fold change ≥1.5 and FDR‐adjusted *p* < 0.05

To investigate the function and mechanisms of BECs in WT and EC‐KO mice of different age groups, we performed RNA‐sequencing with the groups shown in Figure 3A. The four groups were analyzed based on differences in both genotype (EC‐KO versus WT) and age (6–7 months versus 8–12 weeks). We used hierarchical clustering to make transcriptome‐wide, unbiased comparisons of WT and EC‐KO in all groups. Datasets clustered together within the biological replicates and were distinct from those of other groups (Figure 3B). The overlap of all the significant DEGs between different compared groups is shown in a Venn diagram (Figure 3C). The expression of five genes associated with BBB function, brain vessel malformation, or transporters, including *Cyp1b1*, *Map4 k3*, *Tspan18*, *Trf*, and *Maf*, was significantly altered in all compared groups. For instance, Cyp1b1 produces metabolites that can impair endothelial barrier function in vitro, whereas Cyp1b1 inhibition can increase BBB permeability for small molecular tracers in vivo.^26^ Notably, there were fewer DEGs in BECs isolated from 6 to 7‐month‐old WT mice than in BECs isolated from 8 to 12‐week‐old WT mice (Figure 3D), suggesting that 6–7 months might not be enough time to generate a significant difference in BBB function. Considering that the effects of CD147 deficiency extended beyond those of increased age, we performed GO analysis (Figure 3E) of 562 DEGs selected from the gene sets from EC‐KO versus WT mice aged both 8–12 weeks and 6–7 months, except for the DEGs of ages based on the Venn diagram in Figure 3C. We found that 7 of the top 20 significantly enriched GO terms were associated with tube and vasculature biological processes, including vasculature development, blood vessel development/morphogenesis, and tube development/morphogenesis (Figure 3E). GO analysis of the 373 DEGs, selected from the gene sets from 6 to 7‐month‐old versus 8–12‐week‐old mice regardless of genotype, except for the DEGs of CD147 treatment factor, revealed information that was less relevant to BBB function or the CNS (Figure 3C,F).

### 3.4 Effects of CD147 deficiency on BBB integrity in vivo and in vitro

**FIGURE 4:**
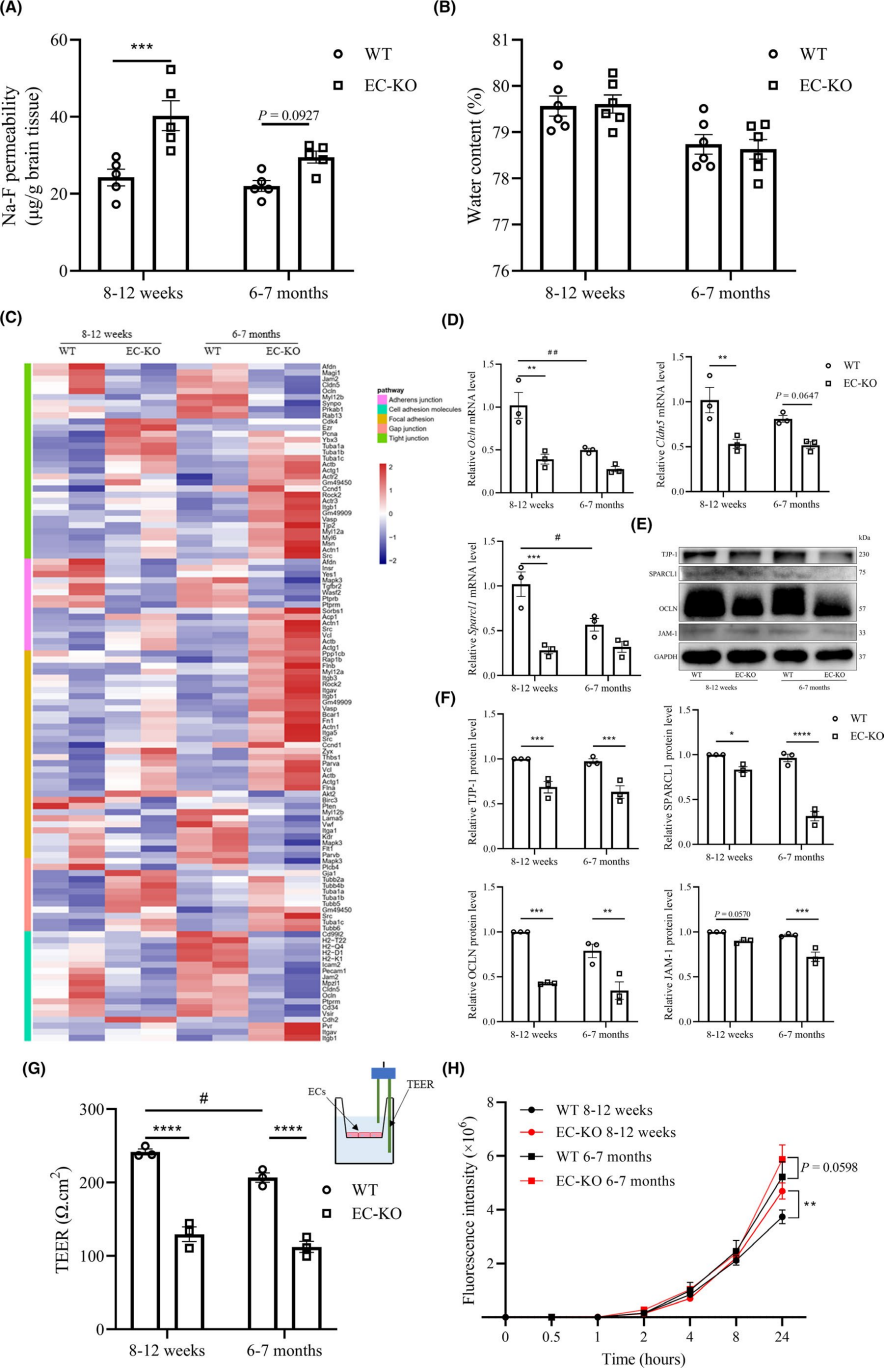
Effects of CD147 deficiency on BBB integrity in vivo and in vitro. (A) WT and EC‐KO mice at 8–12 weeks or 6–7 months of ages were given an i.v. injection of sodium fluorescein (Na‐F), and the absorption of Na‐F in the mouse brain was measured by a microplate reader at 485 nm, *n* = 5. (B) Brain water content of WT and EC‐KO mice at 8–12 weeks or 6–7 months of age, *n* = 6. (C) Heatmap showing the 82 DEGs (see Table S1) from the following five KEGG pathways: Tight junction (ko 04530), Adherens junction (ko 04520), Focal adhesion (ko 04510), Cell adhesion molecules (CAMs; ko 04514) and Gap junction (ko 04540). All these enrichment pathways were *p* < 0.05 by hypergeometric tests. (D) Expression changes of selected genes (*Ocln*, *Cldn5*, *Sparcl1*) from the above dataset by RT‐qPCR. Data are presented as the mean ± SEM of 3 independent experiments. (E) Western blot of DEGs of interest (OCLN, TJP1, SPARCL1, JAM‐1). (F) Quantification of protein levels of molecules in (E) relative to GAPDH. (G) TEER measurement at 7 days after seeding BECs in the upper chamber via an EVOM Volt‐Ohm resistance meter with STX2 chopstick electrodes. A schematic of the in vitro monocellular BBB model and TEER measurement is shown (upper right), *n* = 3 per group. (H) The paracellular permeability of the BBB model was assessed by 40 kDa FITC‐dextran at different timepoints, including 0, 0.5, 1, 2, 4, 8, and 24 h. The fluorescence was measured using a Cytation Imaging Reader, *n* = 3 per group. *p < 0.05, **p < 0.01, ***p < 0.001, and ****p < 0.0001 compared to WT at the same ages; ^#^ *p* < 0.05 and ^##^ *p* < 0.01 compared to 8–12 weeks with the same genotype

To explore the role of CD147 in BBB barrier function, we first evaluated BBB integrity in EC‐KO mice. We found that the permeability of the BBB to sodium fluorescein (Na‐F) was significantly higher in 8–12‐week‐old EC‐KO mice than in littermate controls (Figure 4A). However, the permeability to Na‐F tended to increase in 6–7 month EC‐KO mice compared with that in littermate controls (*p* = 0.0927). To assess brain microedema, we measured the water content in the brain and observed that there was no difference between EC‐KO and WT mice at either age (Figure 4B). As the barrier function of the BBB is mainly dependent on cerebrovascular endothelial TJs and adherens junctions (AJs),^27^ we subsequently analyzed the sequencing datasets. We chose 82 DEGs (excluding the DEGs with FPKM ≤ 10) from five KEGG pathways that were highly relevant to junctions and adhesions. These pathways had enrichment *p*‐values <0.05 via hypergeometric tests (Figure 4C and Table S1). *Cldn5*, one of the best‐known TJ proteins regulating the integrity and permeability of the BBB,^6^ were downregulated in BECs isolated from EC‐KO mice (Figure 4C,D). Other junction‐related proteins, such as *Vwf*, *Ocln*, and *Tjp1*, were also diminished under CD147 deficiency. These results indicated that CD147 had a substantial effect on molecular expression patterns involved in BBB barrier function. Concordant with the sequencing datasets, RT‐qPCR (Figure 4D and S1A) and western blot (Figure 4E,F) assays further validated the reduction in the expression of a subset of DEGs in aged of CD147‐deficient tissues at both the mRNA and protein levels. Additionally, a further analysis using GTEx portal also showed that CD147 was correlated with the genes encoding TJs or AJs (Figure S1B).

Furthermore, we established a monocellular BBB model of mouse primary BECs in vitro and measured its TEER (Figure 4G). The reduction in TEER verified the impaired paracellular tightness underaged or CD147‐deficient conditions, suggesting a critical role of endothelial CD147 in the regulation of paracellular junctions and adhesions (Figure 4G). We also measured the permeability of the monocellular BBB model in vitro with a fluorescence tracer (40‐kDa FITC‐dextran). Paracellular permeability tended to increase under CD147 deficiency in both the 8–12 week (*p* = 0.0080) and 6–7 month (*p* = 0.0598) groups (Figure 4H). Therefore, these results demonstrated that CD147 deficiency as well as aging contributed to impaired BBB integrity in vivo and in vitro.^28^

### 3.5 Effects of CD147 deficiency on the expression of receptors and carriers in the BBB

**FIGURE 5:**
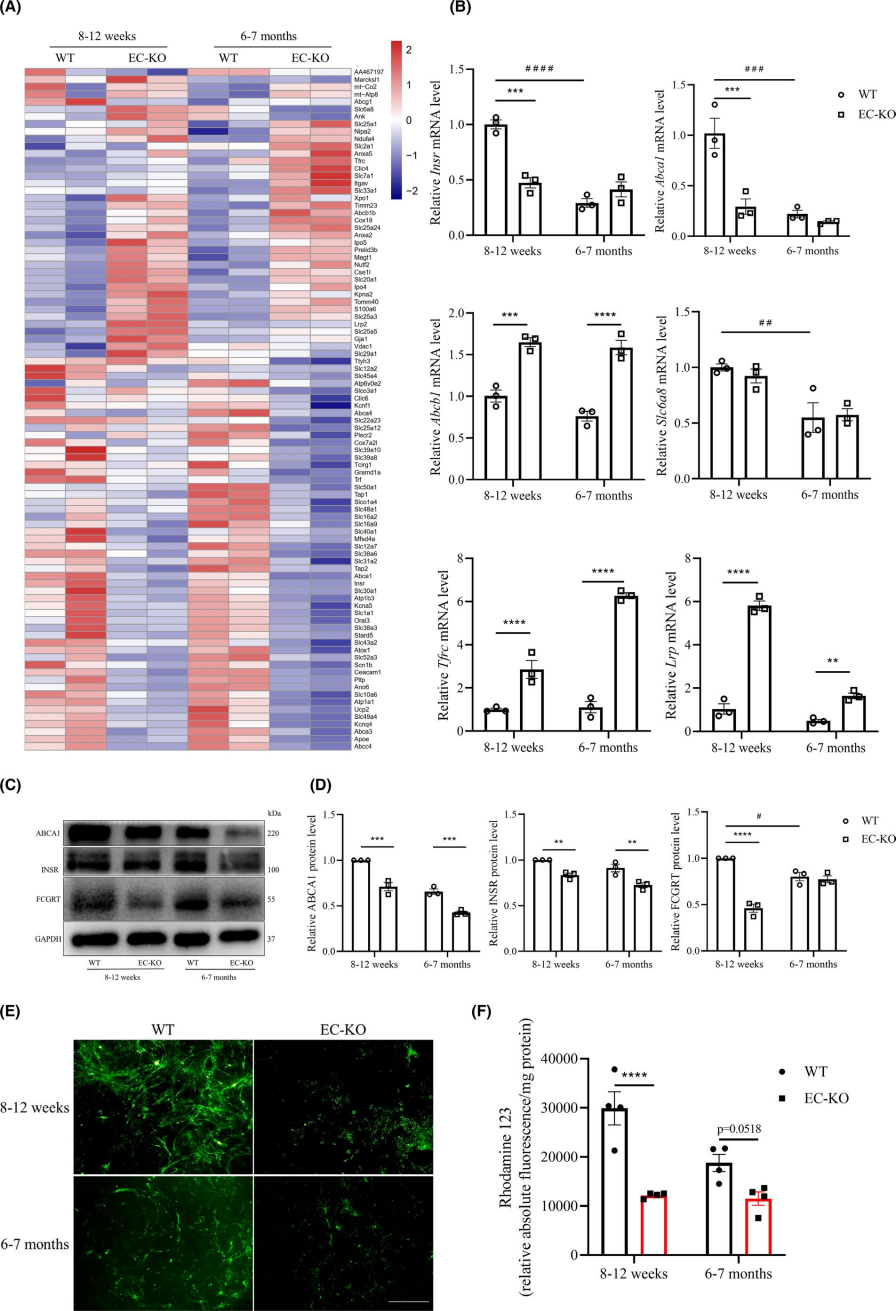
Effects of CD147 deficiency on the expression of receptors and carriers in the BBB. (A) Heatmap showing the 92 DEGs (see Table S1) associated with receptors and carriers. (B) Expression changes of selected genes (Insr, Abca1, Abcb1, Slc6a8, Tfrc, Lrp) from the above heatmap by RT‐qPCR. Data are presented as the mean ± SEM of three independent experiments. (C,D) Western blotting analyses of DEGs of interest (ABCB1, INSR, FCGRT). (E,F) ABCB1 function was assessed by Rho123 uptake assay. Representative images are shown, scale bar: 200 μm (E), and quantitative results are shown (F), *n* = 4 per group. **p < 0.01, ***p < 0.001, and ****p < 0.0001 compared to WT at the same ages; ^#^ *p* < 0.05, ^##^ *p* < 0.01, and ^###^ *p* < 0.001 compared to 8–12 weeks with the same genotype

We next investigated the roles of CD147 in transporter function, another important function of the BBB,^6^ and performed a transcriptome‐wide GO analysis of DEGs in WT and EC‐KO mice. Ninety‐two DEGs (excluding DEGs with FPKM ≤ 5) were enriched (Figure 5A and Table S1). The expression of a variety of well‐known genes encoding receptors and carriers in the BBB, such as *Slc2a1*, *Insr*, *Tfrc*, *Abca1*, and *Abcb1*, was markedly altered (Figure 5A). INSR on the BBB can stimulate transport into the brain and act as a Trojan horse molecule.^29^ Moreover, the mRNA and protein levels of these transporter‐associated genes were confirmed by RT‐qPCR (Figure 5B) and western blot (Figure 5C,D) analyses, which indicated downregulation of *Insr* and *Abca1* and upregulation of *Tfrc*, *Abcb1*, and *Lrp* in BECs isolated from EC‐KO mice. Additionally, GTEx analysis indicated a certain correlation between *CD147* and transporters, such as *Slc2a1*, *Insr*, *Abca1*, and *Slc6a8* on mouse BECs (Figure S1C).

One of the biggest obstacles to the treatment of neurodegenerative disease is that the BBB prevents a majority of macromolecules from being transported from the circulation to the parenchyma.^13^ Several drug delivery systems have been explored to overcome these barriers, including systems that utilize nutrient receptor proteins from the BBB,^11^ engineered with nanoparticles,^12^ liposomal cargoes,^30^ or bifunctional antibodies.^9^ The transferrin receptor (TfR, encoded by *Tfrc*), regarded as the most common transporter to deliver drugs to the brain with clear clinical potential,^9^, ^10^ was increased in both the RNA‐sequencing and RT‐qPCR results (Figure 5A,B). *Abcb1*, which encodes MDR1/P‐gp, is involved in BBB transporter function and Aβ clearance in the brain.^31^, ^32^ As brain uptake is often restricted by the active efflux transporter ABCB1, we evaluated the effect of CD147 ablation on ABCB1 by Rho123 uptake assay.^33^ We found a reduction in Rho123 accumulation in BECs isolated from EC‐KO mice (Figure 5E,F), confirming that endothelial CD147 deficiency resulted in an increase in ABCB1 expression. Remarkably, proteomic analysis and I^125^‐anti‐CD147 brain uptake assays showed that CD147 is a highly expressed BBB protein that could serve as a transporter across the BBB.^18^ Collectively, these findings indicated that CD147 itself and its relevant receptors and carriers play crucial roles in maintaining the transporter function of the BBB.

### 3.6 Disease‐related transcriptomic changes in EC‐KO mice

**FIGURE 6:**
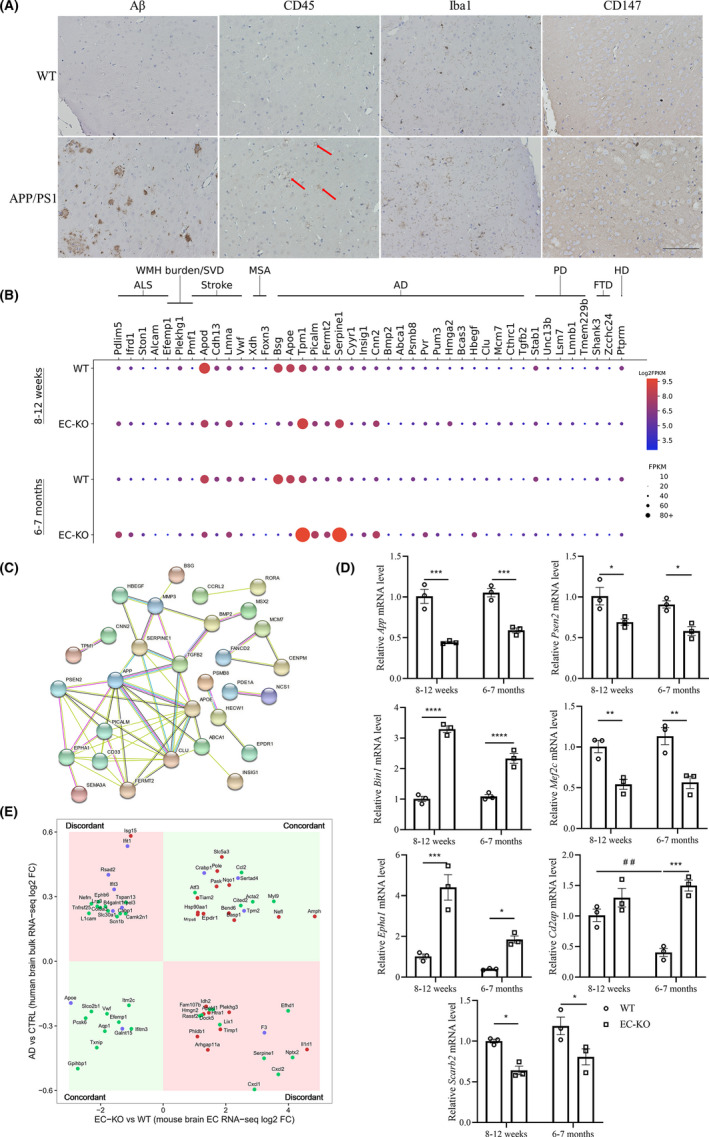
Disease‐related transcriptomic changes in EC‐KO. (A) IHC staining of Aβ, CD45, Iba1, and CD147 in 18‐month‐old APP/PS1 mouse brain slices and peer comparisons. Scale bar: 100 μm (B) The expression of DEGs whose human orthologs are related to neurodegenerative and cerebrovascular diseases in GWAS datasets, expression is represented as FPKM. Disease abbreviations: ALS, amyotrophic lateral sclerosis; WMH burden/SVD, white matter hyperintensity burden and cerebral small vessel disease; MSA, multiple systems atrophy; AD, Alzheimer's disease; PD, Parkinson's disease; FTD, frontotemporal dementia; HD, Huntington's disease. There were 21 DEGs related to AD (*p* = 0.00266). (C) Visual PPI network of the AD‐related dataset. The edges between nodes represent different protein–protein associations, including experimental determination (purple), interactions from current databases (blue), and coexpression (black). (D) Expression changes of selected genes (App, Psen2, Epha1, Cd2ap, Bin1, Mef2c, Scarb2) by RT‐qPCR. Data are presented as the mean ± SEM of three independent experiments. *p < 0.05, **p < 0.01, ***p < 0.001, and ****p < 0.0001. (E) Human AD brain differential expression versus the control (*p* < 0.05) served as the y‐axis, and DEGs (fold change ≥2 with FDR‐adjusted *p* < 0.05) from EC‐KO mice versus WT mice served as the x‐axis. The genes and their human orthologs were concordant in the first and third quadrants (light green), whereas they were discordant in the second and fourth quadrants (light red). Twenty‐one DEGs were specific in the groups compared at 8–12 weeks of age (red dot, EC‐KO versus WT), and 35 DEGs were specific in the groups compared at 6–7 months of age (green dot), whereas 11 DEGs were shared (blue dot) in both groups

A great deal of evidence suggests that inflammation plays a central role in the pathogenesis of neurodegenerative diseases, especially AD.^3^, ^4^ Given that CD147 has been regarded as a promoter of inflammation,^34^ we assessed the expression of CD147 in *APP*/*PS1* transgenic mouse brains by IHC. As shown in Figure 6A, the expression of CD147 was upregulated and CD45^+^ cells and Iba1^+^ microglial cells were activated in AD mice. Previous GWAS analyses identified a series of genes associated with neurodegenerative and cerebrovascular diseases.^35^, ^36^, ^37^, ^38^, ^39^, ^40^, ^41^, ^42^, ^43^, ^44^ We wondered whether several of these genes would have an association with CD147, so we compared the published genes with our sequencing results and found 42 DEGs (FC ≥ 2, FDR‐*P* < 0.05, and excluding DEGs with FPKM ≤10) among the eight diseases (Figure 6B, Table S1). Among these, 21 DEGs were related to AD (FDR‐*P* = 0.00266, five genes overlapped with AD C0002395 on ToppGene DisGeNET Curated). To better understand the relationships of the genes in the AD‐associated dataset (APP and PSEN2^45^ were added for their roles in AD), the PP analysis in the STRING database was employed to generate a visual network (PPI enrichment *p* < 0.0001). Notably, PPI analysis showed that CD147 was closely involved in the intricate network in the AD dataset (Figure 6C). We further validated the DEGs based on the disease‐related dataset via RT‐qPCR (Figure 6D). Intriguingly, we found that the expression of *App* and *Psen2* was decreased after CD147 knockout, which was consistent with the GTEx database analysis (Figure S2A). *Mef2c*, a transcription enhancer factor involved in AD pathological progression^46^ by limiting the brain inflammatory response,^47^ was decreased after CD147 knockout (Figure 6D and S2A). Previous studies suggested that BIN1 expression is increased in the cortex of AD patients.^48^ Our results also showed that *Bin1* expression was increased in CD147‐deficient conditions (Figure 6D). Additionally, *Epha1* and *Scarb2*, which were reported to be involved in AD and PD,^49^, ^50^, ^51^ were verified to be enriched in the absence of CD147. Therefore, all these results indicated that deletion of the BBB protein CD147 was likely to be a strong contributor to the progression of neurodegenerative diseases.

Moreover, we focused on the relationship between DEGs of the mouse cortex after EC‐KO and changes in the homologous genes in human AD brains. We utilized the Allen Brain Institute Aging, Dementia and Traumatic Brain Injury study dataset, which included 111 samples from 30 patients with AD and 58 samples from 16 normal people as controls. There were 67 genes shared with the DEGs in EC‐KO mice (8–12 weeks and 6–7 months) and the changed homologous genes in human AD brains. The changes in expression of several genes were consistent between the two compared groups, whereas some were not (Figure 6E). The possible reason might involve differences in species, brain areas, cell types, and sequencing tools. Specifically, among the 67 DEGs, 11 DEGs shared the same changes in KO mice aged 8–12 weeks and 6–7 months (blue plot), and several of them were functionally related to BBB impairment or neurodegenerative diseases. ApoE, one of the strongest genetic risk factors in sporadic AD, can mediate Aβ clearance from the brain in mice.^1^ Human ApoE has three isoforms, whereas mice express only a single allele.^2^ Notably, ApoE expression was decreased in both human AD patients and EC‐KO mice (Figures 6B,E and S2B). It has been demonstrated that ApoE4 in human PCs damages BBB integrity^52^ and that ApoE4 in glia enhances APP transcription and Aβ generation.^2^ AMPH is a homolog of the AD risk factor BIN1^53^ and was upregulated in EC‐KO mouse brains and human AD patients (Figures 6B and S2B). In summary, transcriptome analysis of CD147‐deficient mouse tissue and tissue from patients with neurodegenerative diseases suggested that CD147 was deeply engaged in the progression of neurodegenerative disease. More evidence regarding the above DEGs needs to be found in specific species, cell types, and other contexts.

## 4 DISCUSSION

In this study, we identified CD147 specific expression on mouse BECs under normal conditions and there was an enormous change with age. To determine the effects of CD147 on the dual roles of the BBB, including its barrier and transporter functions, we generated EC‐KO mice. Primary cortex ECs from mice of different genotypes and ages were isolated and RNA‐seq was performed. CD147 was essential for maintaining BBB integrity and regulating carriers and receptors on the BBB. This study also provides considerable evidence that CD147 plays a critical role in neurodegenerative diseases, particularly AD, which might promote future experimental investigations in neurodegenerative diseases.

Our results indicate that BBB integrity is affected by endothelial CD147 deficiency. EC‐KO mouse brains were more easily permeated by Na‐F, a 376‐Da tracer, than WT mouse brains. However, the brain water contents of WT and EC‐KO mice were comparable, suggesting a limited breakdown of the BBB. The lower TEER and higher permeability of the monocellular BBB model in vitro are also concordant with the results in vivo. We enriched 82 DEGs that belonged to five barrier‐related pathways from KEGG pathway analysis and subsequently validated several important genes and illustrated the correlations between CD147 and these genes. The crosstalk between TJs and AJs in maintaining BBB integrity is a potential research direction to better understand the regulation of barrier function,^27^ and CD147 may be involved in this process under both physiological and pathological conditions. However, further studies are recommended to find the corresponding molecular mechanism to verify these findings.

The receptors and carriers of the BBB are significant for maintaining brain hemostasis by mediating the materials that cross the barrier. On the one hand, brain cells require nutrients, such as glucose and amino acids, for biosynthesis. On the other hand, metabolic products also need to be transported out of the brain. Both processes depend on special receptors and carriers on the BBB.^54^, ^55^ CD147 is one of the potential Trojan horse molecules highly expressed on BECs and its antibodies were detected in the parenchyma after tail intravenous injection.^18^ One of the genes under‐regulated under CD147 deficiency, *Slc2a1*, encodes a major glucose transporter (GLUT1) on the BBB (Figures 5A and S1C). This transporter can deliver glucose from the circulation to the CNS, further participating in energy metabolism.^54^ Other molecules used as transporters, such as TfR, Lrp, and FcRn, have been well studied.^9^, ^10^, ^13^ In particular, ABCB1 expression decreases with age,^56^ and it serves as an efflux pump reducing brain Aβ burden,^31^ which is essential for CNS microenvironment equilibrium.^57^ In addition, the coexpression of CD147 and ABCB1 has also been reported in prostate cancer,^55^ which implies a potential relationship of these proteins in BECs. We analyzed the relevant DEGs regulated by CD147 and found that CD147 was correlated with several of these DEGs. Therefore, CD147 itself as well as its relevant receptors and carriers play crucial roles in maintaining the transporter function of the BBB.

Apart from the maintenance of BBB integrity and the regulation of carriers and receptors, CD147 also provides neuroprotective effects against in vitro oxidative and ischemic neuron injury.^58^ It is remarkabe, however, that CD147 has been reported to contribute to secondary damage after stroke by disrupting the BBB permeability through MMP activation.^19^ In addition, cyclophilin, the ligand for CD147, has been demonstrated to the induction of pericyte‐associated BBB disruption after subarachnoid hemorrhage (SAH) via proteolytic functions for the degradation of endothelial TJ proteins and basal membranes.^59^ Several possible explanations have been offered for the discrepancy of observed CD147 functions in these studies. For instance, CD147 functions may depend on the physiological and pathological processes where the detrimental effects of overexpressed CD147 on the BBB have been investigated in acute ischemic stroke and SAH.^19^, ^60^ Moreover, CD147 expression is significantly increased after stroke and SAH on various cell types, such as infiltrating monocytes, astrocytes, endothelial cells, and pericytes,^59^, ^61^ whereas it is specifically expressed in BMECs in a physiological process.

By a combined analysis of our sequencing data, GWAS, and the Allen Brain Atlas, we investigated promising roles of CD147 in the etiology of neurodegenerative diseases and the relationship between CD147 and other known genes.^35^, ^40^, ^62^ Endothelial *CD147* knockout downregulates the expression of genes, including *App*, *Psen2*, *Apoe*, and *Mef2c*, that are risk factors for familial and sporadic AD. However, several genes are upregulated under CD147 deficiency, such as *Epha1* and *Bin1*, which might indicate an obscure role of CD147 in the course of AD. The probable reason is that AD is a complex disease susceptible to a variety of factors, such as age, genes, inflammation, environment, education, race, and even interpersonal difference.^3^, ^11^, ^40^, ^45^ In particular, IFITM3 plays a regulatory role in γ‐secretase, which facilitates the production of Aβ in neurons and astrocytes,^4^ and we found that CD147 deletion decreases its expression in both mice and humans (Figure S2A,B). In general, endothelial CD147 deficiency reduces the expression of core AD genes, including *App*, *Psen2*, and *Ifitm3*, and we hypothesize that CD147 is a promising risk factor for AD and other neurodegenerative diseases. Further investigation is needed to prove this hypothesis.

CD147 also acts in other contexts, including inflammation,^34^ angiogenesis, vascular remodeling,^60^ and pathogen infection.^14^, ^15^ It has been reported that CD147 overexpression in tumors contributes to angiogenesis by increasing VEGF production via the PI3 K/AKT pathway.^63^ In our study, the expression levels of *Vegfa* and *Vegfd* were altered, and the PI3 K/AKT pathway was also enriched in KEGG analysis (data not shown), which suggests that brain vessel density may be changed by CD147 deficiency, this possibility needs further evaluation. CD147 is also used by meningococci in brain vascular adherence and colonization, which is an inevitable step in the infectious disease,^15^ and this role also implies a possible role of CD147 in adhesion. All these factors are worth investigating during BBB formation and in the corresponding pathological conditions.

We wish to emphasize that our analysis of data from our transcriptomic sequencing and public databases is just an elementary attempt. To find a causal relationship between DEGs and CD147 as well as alterations in the dual functions of the BBB and the roles of CD147 in neurodegenerative diseases, further mechanistic experiments of target gene(s) are required. In this study, BECs were isolated from the whole cortex, which may cover the regional heterogeneity of the brain, and transcriptomic datasets from neurovascular cells or other cells will be necessary to uncover the potential changes in specific brain zones. Further studies will also be considered to examine the differences in sex, cell type, and even more advanced species using similar approaches and analyses, which may provide a clearer understanding of the triangular relationships among CD147, BBB, and neurodegenerative diseases.

In conclusion, this is the first study to propose a triangular relationship among CD147, the BBB, and AD using a range of bioinformatic analyses. Functional experiments based on endothelial CD147 knockout both in vivo and in vitro suggested that CD147 coordinates the dual functions of the BBB, including its barrier and transporter functions, and provided significant insights into the maintenance of BBB homeostasis. Our data also suggest the possibility of targeting CD147 as a promising therapeutic strategy for AD.

## CONFLICT OF INTEREST

The authors declare no conflict of interest.

## Supporting information

Fig S1

Fig S2

Table S1

## Data Availability

The dataset generated and analyzed during the current study is available from the corresponding author on reasonable request. Our RNA‐seq original sequence data have been submitted to the database of the NCBI Sequence Read Archive (http://trace.ncbi.nlm.nih.gov/traces/sra) under the BioProject ID: PRJNA694118.
