# Supplementary figures and images for: Endothelial genetic deletion of CD147 induces changes in the dual function of the blood‐brain barrier and is implicated in Alzheimer’s disease
Source: CNS Neurosci Ther. 2021 May 13;27(9):1048–63. doi: 10.1111/cns.13659 (PMC8339530; doi:10.1111/cns.13659)

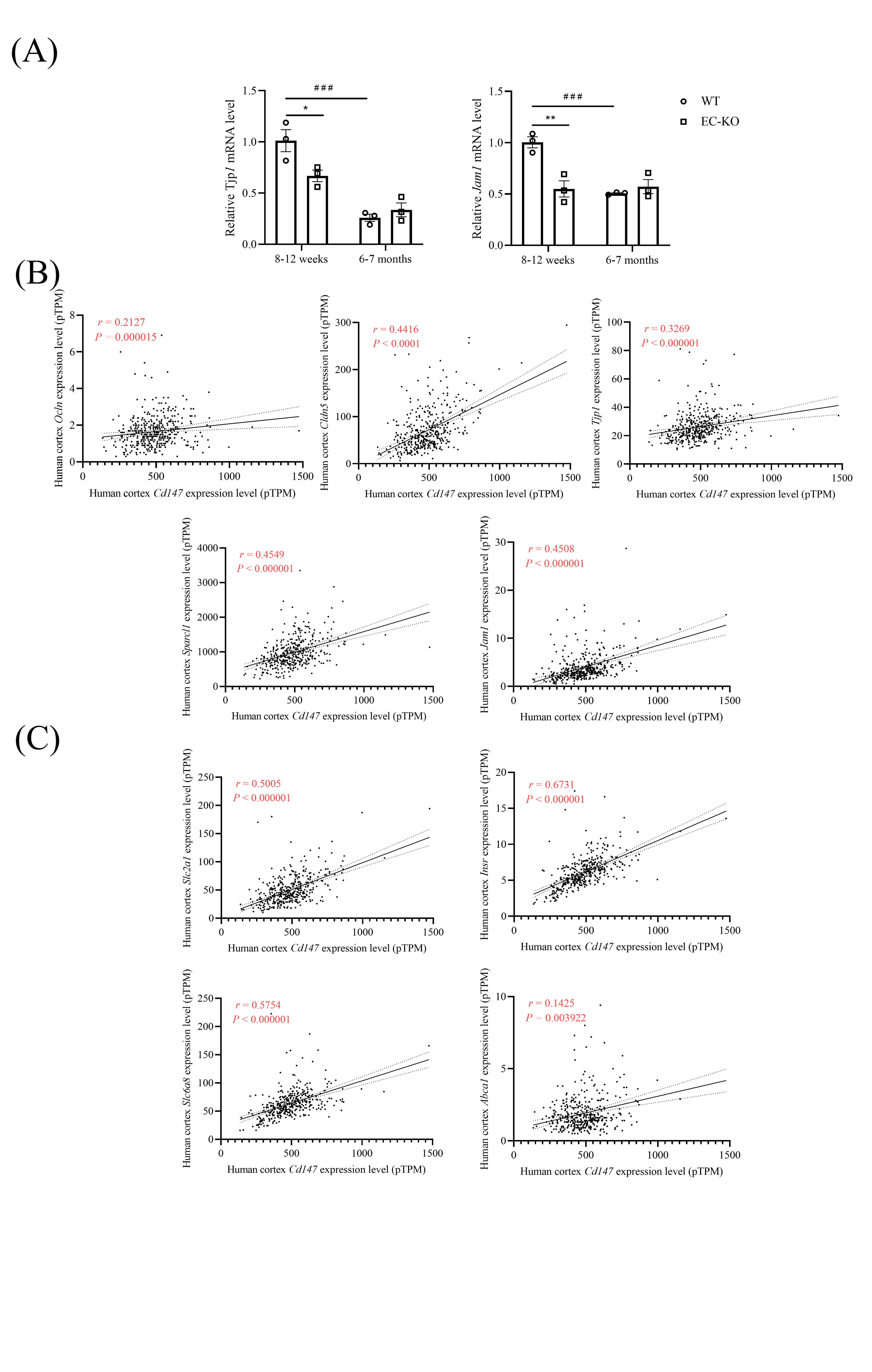

Supplement: Supplementary file 1 — Fig S1 [file CNS-27-1048-s002.TIF]

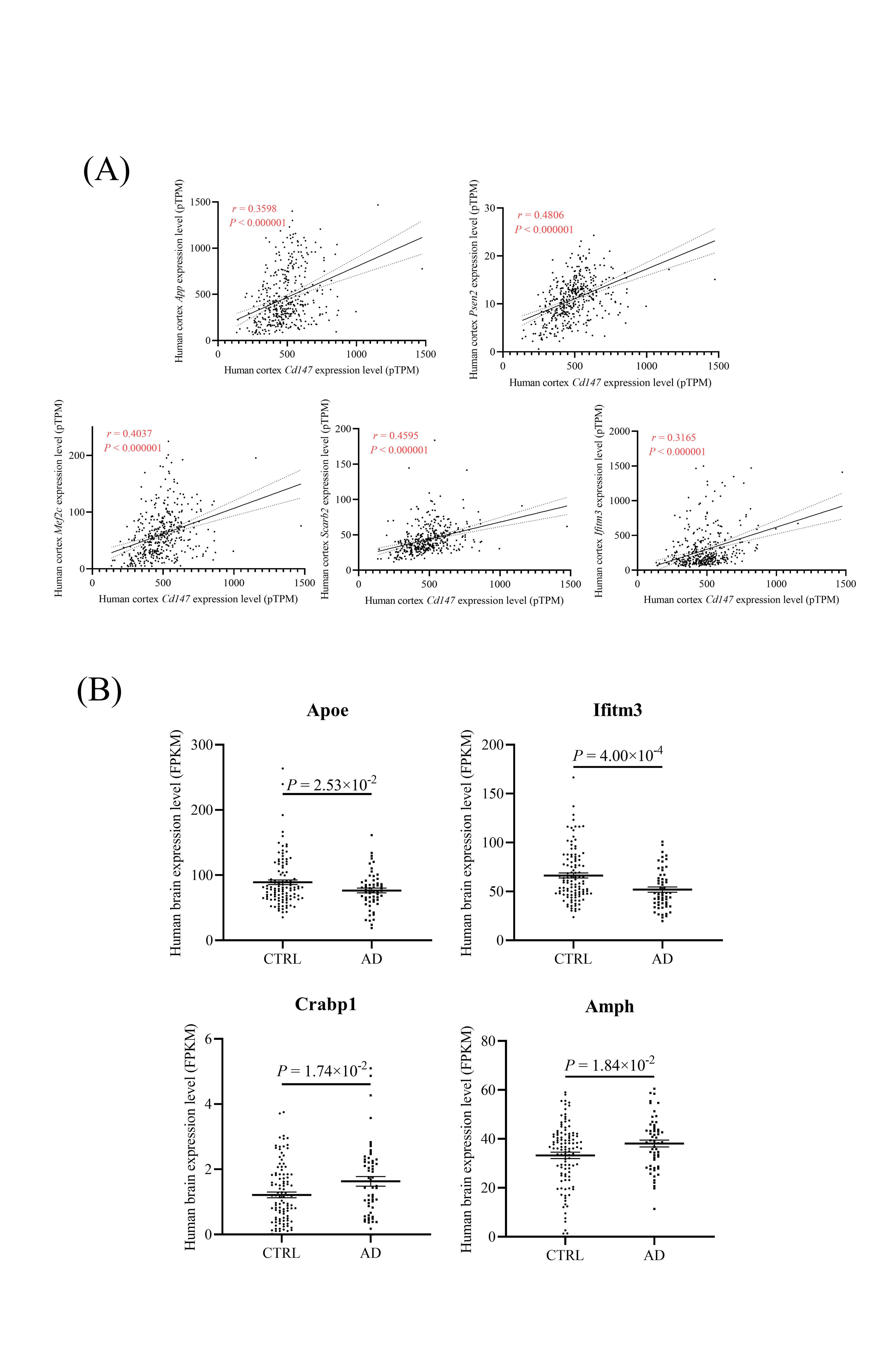

Supplement: Supplementary file 2 — Fig S2 [file CNS-27-1048-s003.TIF]
