# Supplementary material for: Endothelial genetic deletion of CD147 induces changes in the dual function of the blood‐brain barrier and is implicated in Alzheimer’s disease
Source: CNS Neurosci Ther. 2021 May 13;27(9):1048–63. doi: 10.1111/cns.13659 (PMC8339530; doi:10.1111/cns.13659)
Supplement: Supplementary file 3 — Table S1 [file CNS-27-1048-s001.docx]

**Supplementary Table 1**. DEGs datasets in this study.

| **Dataset** | **Gene symbols** |
| --- | --- |
| Barrier | Itga5/Icam2/Tubb6/Vwf/Insr/Ppp1cb/Actn1/Lama5/Cd34/Vsir/Actr2/Ptprb/Rock2/Itgb3/Pecam1/Ocln/Vcl/Parvb/ Myl12a/Tjp2/Sorbs1/Flnb/Itgb1/Fn1/Actr3/Mpzl1/Itgav/Src/Rab13/Wasf2/Prkab1/Actb/Flt1/Vasp/Parva/Msn/Flna/ Bcar1/Tgfbr2/Ptprm/Myl12b/Cd99l2/H2-Q4/Thbs1/Pvr/Cldn5/Itga1/Synpo/Tuba1c/Acp1/Magi1/Rap1b/Jam2/ H2-T22/H2-K1/Actg1/Kdr/Mapk3/H2-D1/Myl6/Gm49450/Gm49909/Tubb5/Akt2/Cdk4/Pten/Yes1/Tuba1b/ Cdh2/Pcna/Zyx/Ybx3/Birc3/Tubb4b/Plcb4/Gja1/Ezr/Tubb2a/Afdn/Ccnd1/Tuba1a/Sparcl1 |
| Transporter | S100a6/Gramd1a/Tcirg1/Ipo4/Cse1l/Tomm40/Apoe/Slc25a1/Nutf2/Slc38a3/Timm23/Abca1/Prelid3b/Slc25a5/Pltp/ Slc12a7/Kpna2/Atox1/Scn1b/Slc6a8/Xpo1/Vdac1/Ank/Tfrc/Slc49a4/Clic6/Slc29a1/Abcg1/Abca3/Cox7a2l/Tap2/ Slc12a2/Slc1a1/Slco3a1/Slc39a10/Slc40a1/Slc25a12/Itgav/Slc20a1/Slc52a3/Anxa5/Slc33a1/Slc50a1/Abca4/ Kcnq4/Slc2a1/Slc10a6/Ndufa4/Slco1a4/Nipa2/Ipo5/Magt1/Anxa2/Plscr2/Atp1b3/Trf/Abcc4/Atp1a1/AA467197/ Ucp2/Slc16a2/Cox18/Ttyh3/Clic4/Tap1/Slc30a1/Slc16a9/Slc43a2/Slc22a23/Atp6v0e2/Slc25a24/Slc7a1/Orai3/ Slc38a6/Kcna5/Stard5/Marcksl1/Gja1/Kcnf1/Slc39a8/Mfsd4a/Slc25a3/Ano6/mt-Co2/mt-Atp8/Slc31a2/Ceacam1/ Slc45a4/Slc48a1/Insr/Lrp2/Abcb1a |
| Disease-related (GWAS) | Pdlim5/Ifrd1/Ston1/Alcam/Efemp1/Plekhg1/Pmf1/Apod/Cdh13/Lmna/Vwf/Xdh/Foxn3/Bsg/Apoe/Tpm1/Picalm/ Fermt2/Serpine1/Cyyr1/Insig1/Cnn2/Bmp2/Abca1/Psmb8/Pvr/Pum3/Hmga2/Bcas3/Hbegf/Clu/Mcm7/Cthrc1/ Tgfb2/Stab1/Unc13b/Lsm7/Lmnb1/Tmem229b/Shank3/Zcchc24/Ptprm |
| AD-related (Allen Brain Atlas) | Amph/Basp1/Bend6/Crabp1/Epdr1/Hsp90aa1/Mrps6/Nefl/Nqo1/Pask/Pole/Sertad4/Slc5a3/Tiam2/Tpm2/Acta2/ Atf3/Ccl2/Cited2/Myl9/Ifit1/Ifit3/Isg15/Rsad2/Slc30a1/Ypel3/B4galnt1/Cabp1/Camk2n1/Col5a3/Ephb6/L1cam/ Lrp3/Nefm/Scn1b/Tnfrsf25/Tspan13/Arhgap11a/Dock5/F3/Hmgn2/Idh2/Il1rl1/Phldb1/Plekhg3/Timp1/Apold1/ Cxcl1/Cxcl2/Efhd1/Fam107b/Htra1/Lix1/Nptx2/Rassf2/Serpine1/Apoe/Galnt15/Aqp1/Efemp1/Gpihbp1/Ifitm3/ Itm2c/Pcsk6/Slco2b1/Txnip/Vwf |
